# Intracranial Translucency, Its Use as a Potential First Trimester Ultrasound Marker for Screening of Neural Tube Defects

**DOI:** 10.3390/diagnostics10110986

**Published:** 2020-11-22

**Authors:** Gerardo Sepúlveda-González, Tayde Arroyo-Lemarroy, David Basurto, Ivan Davila, Esteban Lizárraga-Cepeda, Angel Regino Guerra-de la Garza Evia, Andrea Alcázar-Juárez

**Affiliations:** 1 Medicina y Cirugía Fetal Monterrey, 66267 San Pedro Garza García, Nuevo León, Mexico; ivanvladimir@hotmail.com (I.D.); dr_estebanlizarraga@yahoo.com.mx (E.L.-C.); 2 Hospital Regional Materno Infantil de Alta Especialidad, 66110 Guadalupe, Nuevo León, Mexico; tayde.arroyo@gmail.com; 3 My fetUZ Department of Development and Regeneration, Cluster Woman and Child, Biomedical Sciences, KU Leuven, Herestraat 49, 3000 Leuven, Belgium; davidbasurtodiaz@gmail.com; 4 Iberoamerican Research Network in Obstetrics, Gynecology and Translational Medicine, 11320 Ciudad de México, Mexico; 5 Departamento de Ginecología y Obstetricia, Hospital Universitario “Dr. José Eleuterio González”, Universidad Autonóma de Nuevo León, 64460 Monterrey, Nuevo León, Mexico; 6 Instituto Tecnológico y de Estudios Superiores de Monterrey, 64849 Monterrey, Nuevo León, Mexico; aguerrage@prodigy.net.mx; 7 División de Ciencias de la Salud, Universidad de Monterrey, 66238 San Pedro Garza García, Nuevo León, Mexico; andrea.alcazar@udem.edu

**Keywords:** prenatal diagnosis, intracranial translucency, first trimester, spina bifida, encephalocele, neural tube defects, screening

## Abstract

The objective of the study was to describe a case-series of neural tube defects (NTD) with an abnormal intracranial translucency (IT) detected during the first-trimester ultrasound scan, performed on a low-risk obstetric population in Mexico. Certified Fetal Medicine specialists performed all US scans; the IT was assessed using the mid-sagittal view of the fetal head, which is already systematically used for nuchal translucency and nasal bone evaluation. During the study, we were able to find that eight fetuses had an absence of the intracranial translucency, out of which two were reassessed at 14 weeks′ gestation and IT was normal, six of them were later diagnosed to have an NTD that consisted in spina bifida aperta (*n* = 5) and encephalocele (*n* = 1). Conclusion: As previous studies have shown, IT evaluation during the first-trimester US routine scan may be a useful screening marker for early detection of NTDs.

## 1. Introduction

Neural tube defects (NTD) are the most frequent congenital anomalies affecting the central nervous system [1,2]. The incidence of NTDs is highly variable and depends upon several factors, including ethnic, demographic, and geographic factors, folic acid supplementation and food fortification programs, prenatal screening programs and access to pregnancy termination [1].

The incidence of NTDs is approximately 1 per 1000 pregnancies, and worldwide, nearly 300,000 infants with NTD are born each year [1].

By 2011, the incidence of NTDs in Mexico was 4.9 per 10,000 live births, out of which 75% corresponded to spina bifida [1]. Spina bifida aperta (SBA) is the most common NTD and is the most severe, non-lethal congenital disability. NTDs are the cause of chronic disability in 70,000 to 100,000 individuals in the United States [3].

Historically, screening was first performed by second-trimester ultrasound (US) scan with a sensitivity close to 100% in detecting NTDs when performed by experienced physicians [1]. Classic ultrasound cranial and cerebellar signs such as the lemon and banana signs, first described by Nicolaides et al. have been used ever since the late-eighties, marking the beginning of prenatal NTD diagnosis as we know it nowadays [4,5,6].

During the first trimester, the SBA defect is difficult to diagnose by looking directly for the spinal defect. In 2009, Chaoui et al. concluded that the mid-sagittal view of the fetal face, which is routinely used in first-trimester screening for chromosomal defects between 11.0 and 13.6 weeks of gestation, may also be used for early screening of SBA [7]. The fourth cerebral ventricle is easily recognizable as an anechoic space referred to as IT [7]. Given this, in the absence of the IT, the sonographer should raise the possibility of an underlying NTD and shall grant further and detailed examination of the head and spine [7].

The ability to identify NTDs early in the gestation could aid in decision making, and some cases of SBA could benefit from fetal surgery. Therefore, it should be essential to offer first-trimester screening for NTDs as part of the routine 11–13 weeks scan in all pregnancies.

The present study is a case series of fetuses with absent IT found during the first-trimester US screening from (2011–2019).

**Figure 1:**
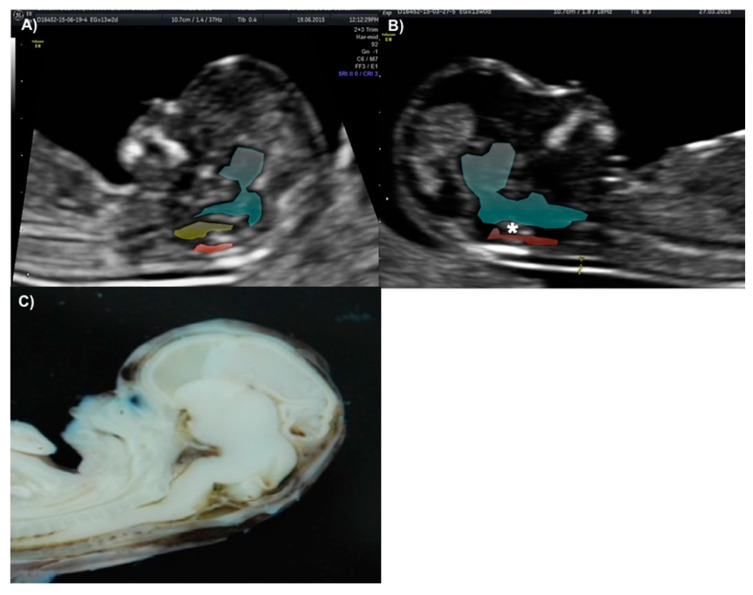
(**A**) Exact mid-sagittal plane of the fetal face at 11—13 weeks scan, showing the nasal bone (NB), nuchal translucency (NT), thalamus, midbrain (MB), brain stem (BS) in green color, cisterna magna (CM) in red color and fourth ventricle in yellow color. (**B**) Mid-sagittal plane of the fetal face showing thalamus, midbrain (MB), brain stem (BS) in green and cisterna magna (CM) in red, the fourth ventricle is missing (*). (**A**) and (**B**) correspond to images obtained from patient presented on case 2. (**C**) Mid-sagittal dissection of a 13 weeks fetus showing anatomic landmarks of the IT, brain stem, fourth ventricle, and CM. (**C**) is displayed courtesy of Ivan Davila, MD, and the embryology department at Universidad Autónoma de Nuevo León.

The technique for IT evaluation was performed under the following criteria: A mid-sagittal view of the fetal head was obtained for routine measurement of nuchal translucency (NT) and nasal bone assessment. The brain stem, fourth ventricle, and cisterna magna (CM) were identified. The fourth ventricle represents the IT, which can be observed as an anechoic structure parallel to the NT and is outlined by two echogenic borders: the dorsal part of the brain stem anteriorly and the choroid plexus of the fourth ventricle posteriorly (Figure 1). The developing cisterna cerebellomedularis generates another thinner translucency, which lies between the fourth ventricle and the occiput [7]. Measurement of the IT was achieved by placing the calipers on the anterior and posterior echogenic borders of the fourth ventricle, similar to that of the NT measurement [8].

## 2. Case Presentation

Between the years 2011 and 2019, we performed 7130 first-trimester routine US scans on low-risk obstetric patients in Mexico. Each ultrasound scan was performed by Certified Fetal Medicine specialists, who assessed the intracranial translucency (IT) using the mid-sagittal view of the fetal head and classified each patient as having IT absence or presence. Ultrasound scans were performed with a General Electrict (GE) equipments, version Voluson 730 expert, Voluson E6 software BT 13 and Voluson E6 software BT16. Eight cases were classified as IT absent, two of them were reassessed at 14 weeks’ gestation, IT was observed and later the diagnosis of spina bifida aperta was ruled out, so these last two cases were not included in the case report below. SBA was confirmed in two fetuses during the first-trimester exam, while the other four were classified as high-risk for NTDs, and the diagnosis was confirmed after the 16th week of gestation.

Local Institutional Review Board approval was not required given the retrospective nature of this observational non-interventional study. Moreover, the paper does not report on primary research. All data analyzed were collected as part of routine diagnosis and treatment. Electronic file review was used for data collection, creating a database in which the names of the patients were not included. All women provided informed consent in case of subsequent use of their anonymized clinical data and records for research purposes.

### 2.1. Case 1

**Figure 2:**
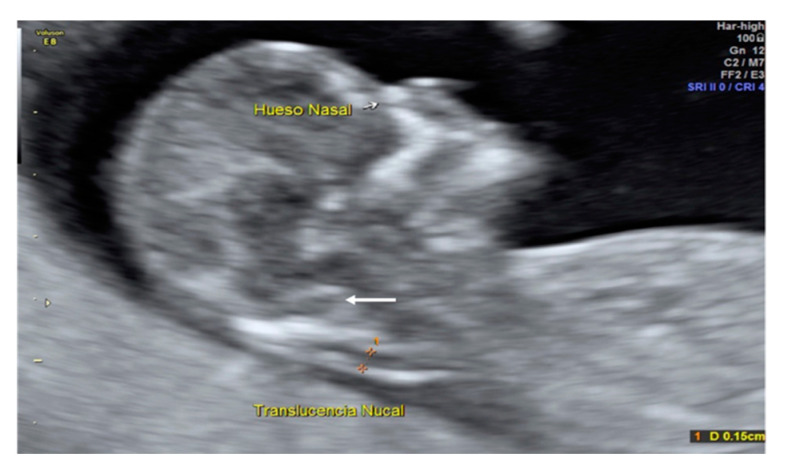
Case 1. First-trimester scan: Mid-sagittal view shows the thickness of the brainstem and a short distance from the posterior border of the brainstem to the occipital bone compared to the brainstem diameter (Big white arrow).

A 35-year-old patient, G3 P2 without relevant medical history was referred for first-trimester screening at 12 weeks of gestation. US findings were a crown–rump length (CRL) of 63 mm, NT of 1.5 mm, present nasal bone, and IT absence (Figure 2). The brainstem was thick, and the distance from the posterior border of the brainstem to the occipital bone was shorter than the brainstem diameter. No US markers for chromosomal anomalies were present. At 16 weeks′ gestation, the diagnosis of lumbosacral SBA was made. After medical counseling, the patient opted for pregnancy termination.

### 2.2. Case 2

**Figure 3:**
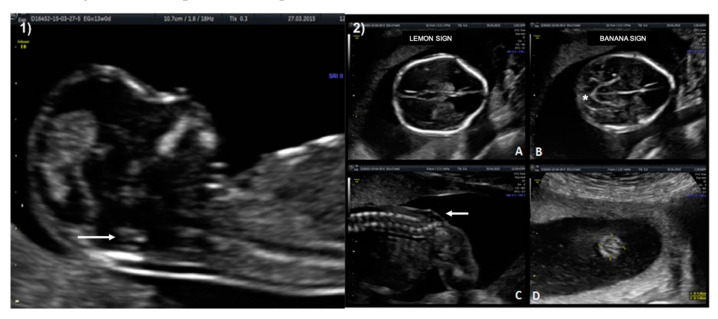
Case 2. First-trimester scan: (**1**) Ultrasound image in the mid-sagittal plane of the fetal face in a case of spina bifida aperta demonstrating compression of the fourth ventricle with no visible IT (white arrow). Second-trimester scan: (**2.A**) Lemon sign on axial plane at 19 weeks. (**2.B**) Banana sign in second-trimester scan (*), (**2.C**) Sagittal plane of the spine shows the neural tube defect between L5-S1 (white arrow). (**2.D**) Axial image from the sac.

A 33-year-old patient G3 P2 without relevant clinical history was referred for routine first-trimester screening at 13 weeks of gestation. The fetus′s CRL was 63 mm, NT measured 1.5 mm, nasal bone was present, and IT was absent (Figure 3-1). A follow-up US scan was performed at 19.4 weeks of gestation. Both the “lemon” and “banana” signs were present (Figure 3-2.A,2.B), and the diagnosis was confirmed by observing an open defect between L5–S1 (Figure 3-2.C). After counseling, the patient opted for expectant management and postnatal repair.

### 2.3. Case 3

**Figure 4:**
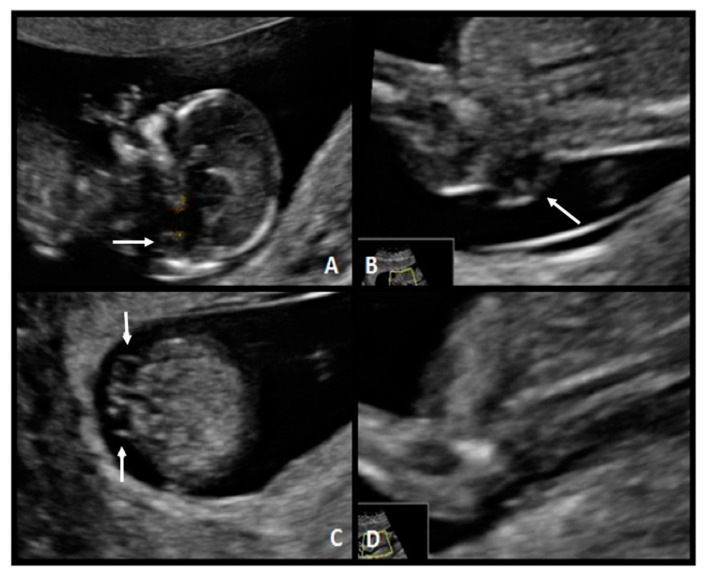
Case 3. First-trimester scan: (**A**) mid-sagittal plane of the fetal face demonstrates compression of the fourth ventricle with no visible IT (white arrow). (**B**) Sagittal view of the lower back with a lumbar spine defect (white arrow) (**C**) axial plane of the spine shows the myelomeningocele (white arrows) (**D**) Axial image.

A 31-year-old, G1 healthy woman was referred for first-trimester screening at 13.1 weeks of gestation. The fetus had a CRL of 76.0 mm, NT was 1.4 mm, the nasal bone was present, and the IT absent. Diagnosis of spina bifida was suspected, and she was classified as a high-risk patient. The US scan at 18 weeks of gestation showed the typical “lemon” and “banana” signs. A lumbosacral SBA was observed (Figure 4). After counseling, this patient opted for fetal surgical repair.

### 2.4. Case 4

**Figure 5:**
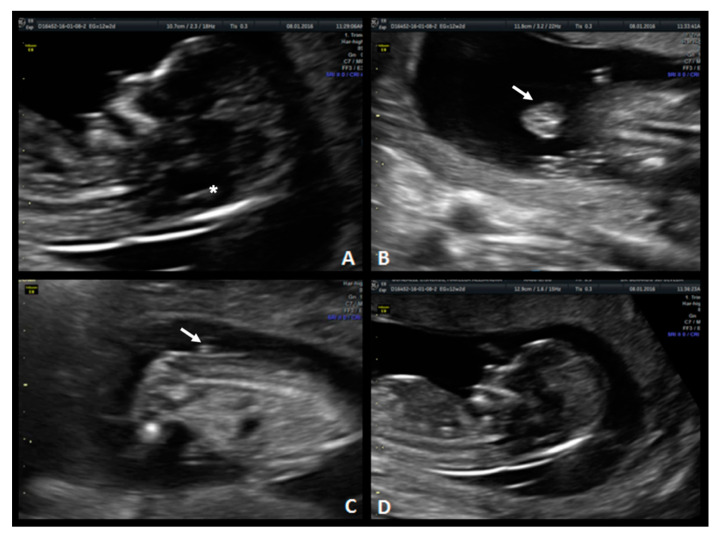
Case 4. (**A**) Mid-sagittal plane of the fetal face demonstrates compression of the fourth ventricle with no visible IT (*). (**B**) Ultrasound view of the lower back with a lumbar spine defect (white arrow). (**C**) Sagittal plane of the spine shows the neural tube defect (white arrow). (**D**) No evident IT.

A 37-year-old woman G5 P3 with a previous daughter with Down syndrome was referred at 12.2 weeks of gestation. The fetus had a CRL of 66.5 mm, NT of 1.4 mm, present nasal bone, and absence of the IT. The diagnosis of spina bifida was suspected and confirmed at 21 weeks (Figure 5). A sacral (S1–S4) SBA was observed. After multidisciplinary counseling, the couple decided on postnatal surgical repair.

### 2.5. Case 5

**Figure 6:**
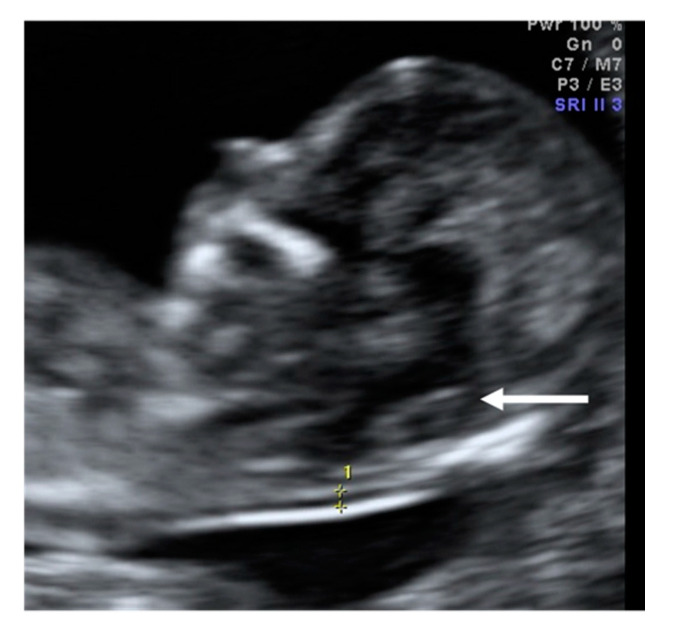
Case 5. Mid-sagittal plane of the fetal face to assess nuchal translucency where IT cannot be identified. The white arrow indicates the place where we should measure IT.

A 34-year-old woman G5 P3 with a history of a daughter with Down Syndrome was referred at 13.2 weeks of gestation. The fetus had a CRL of 72 mm, NT was 1.2 mm, the nasal bone was present, and the IT was absent (Figure 6). Diagnosis of spina bifida was suspected. The patient returned to our center at 21 weeks of gestation, and the US showed the typical “lemon” and “banana” signs and a lumbosacral SBA. After counseling, the patient opted for postnatal surgical repair.

### 2.6. Case 6

**Figure 7:**
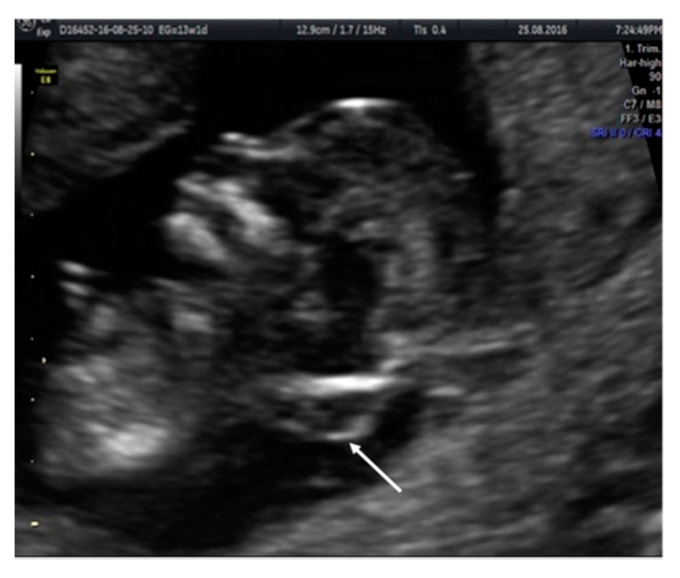
Case 6. The absence of intracranial translucency (IT) and the increase of the brain stem are observed. Additionally, an occipital encephalocele (white arrow) can be observed.

A 30-year-old woman G2 P1 with a history of insulin resistance was referred for first-trimester screening at 12 weeks gestation due to an increased NT. The fetus had a CRL of 56 mm, NT was 2.5 mm, the nasal bone was present, and the absence of the IT was documented. Additional findings included tricuspid regurgitation and suggestive images for congenital heart disease, single umbilical artery, and encephalocele (Figure 7). Due to these findings, the patient was classified as having a high risk for chromosomal anomalies. Invasive diagnostic tests were offered to confirm the suspicion, but the patient opted for termination of pregnancy.

## 3. Discussion

In this case series, we present eight cases with an abnormal IT at the first trimester US scan, among which six fetuses had confirmation of an NTD. Measurement of the IT has been previously described as a useful US marker for the early detection of NTDs [7] along with other markers which are listed below for a wholesome comparison regarding their utility as early-detection tools while performing the first-trimester routine scan.

Since the implementation of the first-trimester screening program for NTDs, we were able to identify all the NTD cases with a 25% false-positive rate. Based on previously published studies, our group considers that the absence of the 4th ventricle—which translates into IT absence on US—is a good first-trimester marker for open NTDs. Given this, sonographers should be appropriately trained and encouraged to assess this structure during the evaluation of NT in the first-trimester US scan. (Figure 1) [9].

There has been widespread acceptance of performing routine US during the first trimester of pregnancy for the past 25 years; given this, in the United Kingdom, the National Screening Committee recommends that this analysis shall be offered to every pregnant woman [10]. Every year, in Mexico, more patients undergo first trimester US for congenital malformations (structural, chromosomal, and genetic abnormalities). IT evaluation grants an inexpensive and non-invasive screening method for detecting NTDs and may be available to the general population [9].

Different authors have studied the direct correlation between the measurement of IT and CRL. In 2016, Molina-Giraldo et al. established a nomogram for the IT in a Latin-American population. [11] They reported a median IT of 1.7 mm (interquartile range, 1.42–2 mm) in fetuses with a CRL between 45 and 84 mm, which is the measurement used to determine IT presence or absence.

Throughout time, several ultrasonographic findings and signs have been proposed to be used as NTDs markers while performing a first-trimester routine US scan [7,12,13,14]. One of these markers was proposed by Finn et al. in 2011, where they measured and recorded the distance between the posterior border of the Sylvian aqueduct and the anterior border of the occiput; in that study, they observed that a shorter distance between these two structures correlated with the incidence of neural tube defects [12]. In another study, Buisson et al. described two cases in which abnormal cerebral peduncles appeared parallel to each other and retracted frontal bones, which resulted in an acorn shape head in fetuses with spina bifida aperta [13]. Lachman et al. proposed evaluating the frontomaxillary facial angle during the first-trimester ultrasound for the screening of open spina bifida; they found that 90% of the fetuses with spina bifida aperta had a lower frontomaxillary angle when compared with controls [14].

Another marker that is evaluated in the mid-sagittal plane used for nuchal translucency is the diameter of the brainstem, which is increased in size by herniation of the posterior fossa in cases of open spina bifida [15]. Lachman et al. found that up to 96.7% of fetuses with spina bifida aperta present an increase in the diameter of the brainstem, so as much as observing the decrease in the fourth ventricle (IT) as observing the increase in the brainstem diameter could contribute to the early diagnosis of spina bifida aperta [15]. This statement correlates with our proposal regarding IT absence as a potential early marker for NTD.

Ever since 1992, the mid-sagittal view of the fetal face has been a part of the routine assessment in every fetus at the 1113– weeks’ scan [16]. Given this, Chaoui and Nicolaides suggested that this view should be used to visualize the posterior brain region. Whenever abnormalities are seen in the diameters of the posterior fossa, fetuses shall undergo an additional assessment by using other planes and techniques to examine the fetal spine [17].

We consider of great relevance the need to emphasize the growing value of prenatal diagnosis of NTDs regarding the future advances on in-utero therapeutic options; given this, we state that one of the great benefits from finding and supporting new early ultrasound signs which aid in early detection of these abnormalities may result in better outcomes for fetuses with spina bifida aperta if they are surgically intervened early in the gestation. The previous statement is supported by Peralta et al., who earlier this year concluded that a lower rate of postnatal complications may occur if the intervention is performed at earlier gestational age, aided by timely detection of the defect [18].

Based on our experience, false positives may arise when the CRL is below 50 mm because at that moment in gestation it may be difficult even for experienced sonographers to identify and limit the three structures we need to look for in the posterior fossa, which are the brainstem, fourth ventricle and cisterna magna. Given this, whenever this situation presents itself, we suggest our patients undergo a second assessment when the CRL approaches 84 mm since this may improve the performance of IT as a marker for spina bifida aperta.

Like others, we found that IT evaluated in the mid-sagittal plane of the fetal face can be used to detect NTDs. However, we acknowledge that no conclusions can be made from our type of study regarding the prognostic value of the marker; hence no recommendations should be made.

## 4. Conclusions

A first-trimester screening program for NTDs was implemented in Mexico. IT is easy to identify in the midsagittal plane. Among the eight abnormal IT cases, five had SBA, one encephalocele, and two were normal.
